# Jejunogastric intussusception: a case report of rare complication of gastrojejunostomy

**DOI:** 10.1093/jscr/rjaa612

**Published:** 2021-07-08

**Authors:** Narjes Mohammadzadeh, Mohammad Ashouri, Sepehr Sahraian, Reza Taslimi

**Affiliations:** Department of Surgery, Imam Khomeini Hospital, Tehran University of Medical Sciences, Tehran, Iran; Department of Surgery, Imam Khomeini Hospital, Tehran University of Medical Sciences, Tehran, Iran; Department of Surgery, Imam Khomeini Hospital, Tehran University of Medical Sciences, Tehran, Iran; Department of Internal Medicine, Imam Khomeini Hospital, Tehran University of Medical Sciences, Tehran, Iran

## Abstract

Jejunogastric intussusception (JGI) is a rare complication of gastrojejunostomy surgery (<0.1% of cases), yet requires an urgent diagnosis. Mortality rate ranging from 10% to 50% based on delay in diagnosis and surgical intervention. Vomiting, abdominal pain and hematemesis are the most common symptoms. We report a 60 years old man admitted to the emergency department, complaining of epigastric pain and recurrent hematemesis for 3 days. Emergent upper GI endoscopy was done, and gastroenterologist reported a protruded edematous jejunal mucosa with bleeding, which formed a mass-like lesion. Abdominopelvic computed tomography scan also showed a target sign in favor of jejunal intussusception. Midline laparotomy and reduction of jejunal loop was performed and the patient was discharged without any further complications. In patients presented with hematemesis and abdominal pain and history of gastrectomy, JGI must considered as a possible cause because early diagnosis and treatment are necessary to prevent further complications.

## INTRODUCTION

Jejunogastric intussusception (JGI) is rare complication of gastrectomy (incidence of 0.15%) and has fatal outcomes if not treated. It is rare but associated with high mortality rates, up to 50% if the diagnosis and treatment are delayed. It can be presented with various clinical presentations including acute and chronic obstruction symptoms [1], hematemesis, intermittent epigastric pain and palpable epigastric mass [2]. The diagnosis can be made based on the clinical presentations, endoscopic findings and imaging. The definitive treatment of JGI is surgical intervention but endoscopic and laparoscopic intervention also can be used. We report a male patient with history of gastrectomy 12 years ago admitted with hematemesis and vomiting and report of gastric tumor with obstruction in further investigation computed tomography (CT) scan and repeated endoscopy diagnosed JGI. Emergent laparotomy resulted in successful treatment.

## CASE PRESENTATION

**Figure 1:**
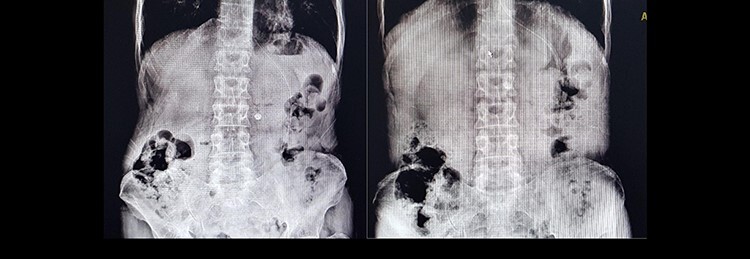
Supine and upright abdominal X-ray.

A 60-year-old male presented to the emergency department complaining of epigastric pain, recurrent hematemesis and coffee-ground vomiting for 3 days. He did not have any history of melena and weight loss. Twelve years earlier, he had undergone distal gastrectomy with vagotomy due to a noncancerous peptic ulcer. He had the endoscopic result with him, which reported gastric tumor with bleeding and obstruction in the gastrojejunostomy site. In physical examination, he was pale and ill. The blood pressure was 80/60 mm Hg, pulse rate at 110 bpm, respiratory rate of 25 per min and he had a temperature of 37°C. In abdominal examination, midline abdominal scar of a previous laparotomy was noted. He had moderate to severe tenderness of epigastric region without guarding, and a mild generalized tenderness without rebound tenderness or mass-like lesion. Bowel sounds and rectal examination were normal. According to our findings, we suspected a gastric tumor as the main cause, and we requested a repeat of endoscopy. Also, upright and supine abdominal X-ray has been taken (Fig. 1).

**Figure 2:**
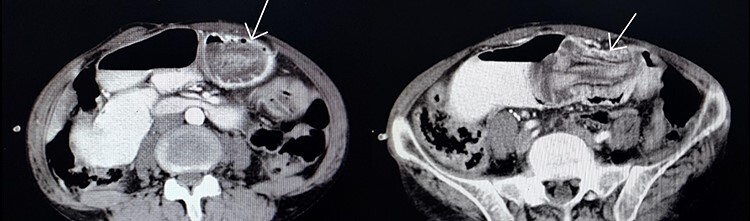
Jejunogastric intussusception showed (arrow) in abdominopelvic CT scan with Iv/oral contrast.

Gastroenterologist reported a 400 cc bloody-biliary discharge in the gastric cavity. A protruded edematous jejunal mucosa which formed a mass-like lesion was also evident. No sign of gangrene or ulceration was reported. In the next step, we requested an abdominopelvic CT scan (Fig. 2) which also showed target sign in favor of intussusception of jejunum. After initial resuscitation, emergency laparotomy was undertaken and in exploration, a distal jejunum loop about 10 cm long, invaginated to the gastric cavity with no sign of ulceration or necrosis was found. The jejunal loop had no pathological causes (leading point) and was reduced without a need for resection. The abdominal cavity was explored entirely, there were no free abdominal fluid or any pathologic finding. Postoperative follow-up was uneventful. The patient discharged 6 days later.

## DISCUSSION

JGI is a rare condition which one or two segments of jejunum invaginate into the stomach from the stoma site of gastrojejunostomy. According to Shackman’s classification It has three types based on the invaginated segment of jejunum: afferent loop, efferent loop or both. The efferent loop invagination is most prevalent [3]. In most reports it occurred at the gastrojejunal anastomosis site and Braun anastomosis site of Billroth II reconstruction, and Y anastomosis site of the Roux-en-Y reconstruction. JGI occurrence after gastric surgery varies from a few days to 25 years postoperatively [4].

The JGI can be categorized based on acute or chronic phase of onset. In acute phase, it presents with colicky pain that can be associated with vomiting, however in some cases other symptoms like epigastric mass and hematemesis are also seen. In chronic type, it can be presented with obstruction symptoms namely chronic intermittent epigastric pain for years [5]. In some articles, epigastric mass with hematemesis and colicky epigastric pain was defined as the pathognomonic triad for JGI. Therefore, it is crucial to differentiate JGI from bleeding ulcer, tumoral lesion and mechanical obstruction due to anastomotic stenosis, bezoar impaction, afferent loop syndrome and other causes. Based on the time of diagnosis and without immediate intervention, the mortality rate could be 10–50% [6].

The predisposing factors for JGI are excessive lifting of the jejunal stump, excessive peristalsis, the diameter of anastomosis orifice, length of the jejunal stump, hyper acidic state, length of afferent loop, jejunal spasm associated with abnormal bowel motility, intra-abdominal hypertension and retrograde peristalsis [7]. There are also contradictory data about initial choice of diagnostic measures; some articles advice endoscopy and others recommend abdominal CT scan or ultrasonography. Although, in most of cases both of them were performed. Endoscopy is the first step to evaluate upper GI bleeding, but there are cases which intussusception was confounded by a clot, tumor or bezoar, exemplified in our case. The typical CT finding of intussusception is a soft tissue mass with a ‘sausage’ or ‘target’ appearance [8]. Treatment is nearly always surgical. In some cases, the reduction of the involved segment, and in others resection of concerning sites for recurrence were the choice of management. However, due to a lack in follow-ups, the recurrence rate of JGI remains unknown. Both laparoscopic and laparotomy reduction approaches have been performed successfully. There are also reports of endoscopic reduction for JGI [9]. This is obvious that a definitive treatment of JGI is the surgical intervention, which includes reduction with correction, resection of involved bowel and revision of the anastomosis [10].

## CONCLUSION

This case and other cases with similar clinical presentation with history of gastric surgery, especially after Billroth II, highlights that hematemesis associated with abdominal pain (showing ischemic mucosa) needs an emergent surgical consultation. The surgical intervention must not be delayed for endoscopic or other investigation, although they may be helpful for diagnosis or treatment planning.

## References

[1] Alhaj Saleh A, Slate R, Habrawi Z, Aryaie A. Jejuno-gastric intussusception: a case report of unusual cause of food intolerance after roux-En-Y gastric bypass. Int J Surg Case Rep 2018;45:126–9. 29605777 10.1016/j.ijscr.2018.03.029 PMC6000905

[2] Loi C-M, Huang S-Y, Chen Y-D, Chen S-D, Wu J-M, Chen K-H. Retrograde jejunogastric intussusception: a case report and review of the literature. Asian J Surg 2014;40:309–12. 24938859 10.1016/j.asjsur.2014.04.001

[3] Ravirajendran S, Munnamgi S, Abdul A. Antegrade jejunojejunal intussusception inside a retrograde jejunogastric intussusception (double intussusception)—a rare case report. Int J Surg Case Rep. 2017;39:264–6. 28881334 10.1016/j.ijscr.2017.08.036 PMC5587876

[4] Kawano F, Tashiro K, Nakao H, Fujii Y, Ikeda T, Takeno S, et al. Jejunogastric intussusception after distal gastrectomy with roux-en-Y reconstruction: a case report. Int J Surg Case Rep 2018;44:105–9. 29499512 10.1016/j.ijscr.2017.12.042 PMC5910517

[5] Tokue H, Tsushima Y, Arai Y, Endo K. Jejunogastric intussusception: life-threatening complication occuring 55 years after gastrojejunostomy. Intern Med. 2009;48:1657–60. 19755769 10.2169/internalmedicine.48.2115

[6] Ho C-Y, Chang C-W, Lee F, Tsai C-H, Lin W-C, Chen M-J, et al. Jejunogastric intussusception presented with hematemesis: a case presentation and review of the literature. Adv Dig Med. 2017;4:35–8.

[7] Kitasato Y, Midorikawa R, Uchino Y, Saku S, Minami T, Shirahama T, *et al*. A case of retrograde intussusception at Roux-en-Y anastomosis 10 years after total gastrectomy: review of the literature. Surg Case Rep. 2016;2:123. 27813022 10.1186/s40792-016-0250-6 PMC5095090

[8] Hammond N, Miller FH, report - DMC. Intussusception into the enteroanastomosis after billroth II gastrectomy and roux-en-Y jejunostomy: sonographic and CT findings. Am J Roentgenol 2001;177:624–6. 11517058 10.2214/ajr.177.3.1770624

[9] Toth E, Arvidsson S, Thorlacius H. Endoscopic reduction of a jejunogastric intussusception. Endoscopy 2011;43:E63. 21287456 10.1055/s-0030-1256103

[10] Lee S, Kwon I, Ryu S, Sohn S. Jejunogastric intussusception: a rare complication of gastric cancer surgery. Int J Clin Exp Med 2014;7:4498–502. 25550976 PMC4276234

